# Pancreatic Adenocarcinoma With Synchronous Colonic Metastases

**DOI:** 10.14309/crj.0000000000000299

**Published:** 2020-01-07

**Authors:** Rohan Yewale, Banumathi Ramakrishna, Kavita Vijaykumar, Partheeban Balasundaram, S. Arulprakash, Patta Radhakrishna, B.S. Ramakrishna

**Affiliations:** 1 Department of Medical Gastroenterology and Hepatology, SRM Institute for Medical Science Hospital, Chennai, India; 2 Department of Histopathology, SRM Institute for Medical Science Hospital, Chennai, India; 3 Department of Surgical Gastroenterology, SRM Institute for Medical Science Hospital, Chennai, India

## Abstract

Metastases from pancreatic malignancy are commonly known to occur in the regional lymph nodes, liver, lung, and peritoneum. Synchronous or metachronous metastasis from the pancreas to the colon is rare, with only 6 cases reported in the literature. We report a man who was found to have adenocarcinoma on biopsies from synchronous lesions in the colon and the pancreas. The immunohistochemistry report revealed the diagnosis of a primary pancreatic malignancy with synchronous colonic metastases.

## INTRODUCTION

Pancreatic cancer is the fourth leading cause of cancer deaths worldwide, with an increasing overall incidence as per data from the Surveillance, Epidemiology and End Results Program registries.^1,2^ A large percentage of affected patients with lesions in the distal body or tail of the pancreas have nonspecific symptoms, and the disease is often detected in its advanced stages when the patient has already developed metastases.^3^ Metastasis commonly occurs in the regional lymph nodes, liver, lung, and peritoneum. Pancreatic cancer with colonic metastasis is a rare entity, with only 6 cases being reported to date in the literature of which 3 were metachronous lesions.^4–9^ We present a man who was diagnosed to have adenocarcinoma on colonic biopsies, which was later proved to be a metastatic lesion originating from the pancreas.

## CASE REPORT

A 71-year-old man presented with complaints of intermittent pain in the right lower abdomen for 6 months. The pain was moderate in intensity and nonradiating. It was associated with poor appetite, early satiety, constipation, and unintentional weight loss of 10 kg over the last 6 months. There was a strong family history of malignancy among his siblings, with 2 brothers dying of unspecified malignancy and a younger sister recently operated for breast malignancy. General and abdominal examination were unremarkable except for pallor. Baseline investigations were essentially normal except for low serum hemoglobin (9.7 g/dL), elevated C-reactive protein (78.5 mg/dL), low serum iron (22 μg/dL), and elevated total iron-binding capacity (272 μg/dL).

**Figure 1.**
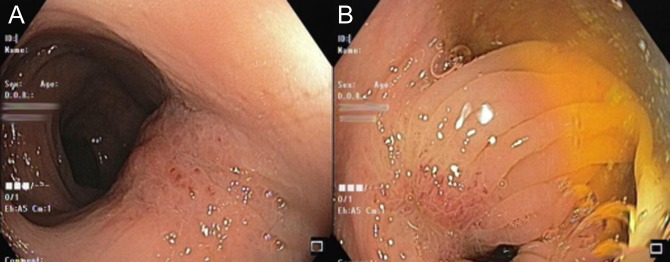
Colonoscopy showing focal scarring and puckering of mucosa in the descending and sigmoid colon mimicking the appearance of healed ulcers.

Further evaluation included a diagnostic upper gastrointestinal endoscopy which was normal and a colonoscopy which showed multiple discrete lesions, separated by normal mucosa, in the descending and sigmoid colon with focal scarring and puckering of the mucosa, suggestive of tumor infiltration from the serosa (Figure 1). The lesions were fleshy and varied in consistency from soft to firm in different affected areas.

Histopathological examination from biopsies of the mucosal lesions (sampled and examined separately from the sigmoid and descending colon) revealed moderately differentiated adenocarcinoma in 2 of the 3 sampled sites. The tumor was composed of irregular and fused glandular structures lined by malignant columnar epithelial cells, and in one fragment, the tumor was seen in the submucosa surrounded by desmoplastic stroma.

Abdominal computed tomography (CT) with contrast showed a heterogeneously enhancing soft tissue density lesion in the body and tail of the pancreas. Serum carcinoembryonic antigen level was normal, and carbohydrate antigen 19-9 level was markedly elevated (1,513 U/mL).

**Figure 2.**
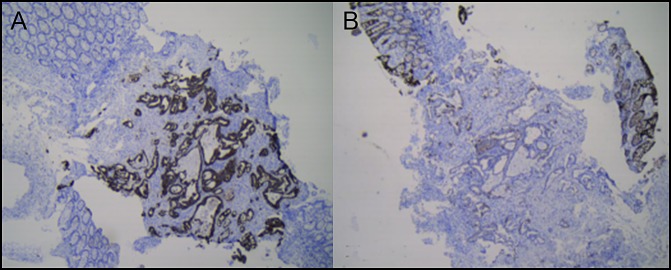
Colonic biopsy. Neoplastic glands (A) showing diffuse strong positive staining for CK7 and (B) are predominantly negative with only a few tumor cells showing positive staining for CK20 (40×).

**Figure 3.**
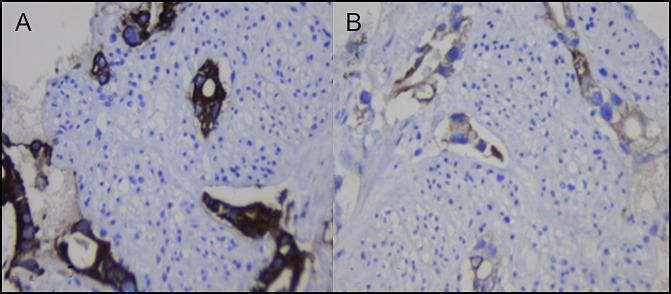
Fine-needle aspiration biopsy of the pancreas. Neoplastic glands showing (A) diffuse strong positive staining for CK7 and (B) focal positive staining in a few tumor cells for CK20 (400×).

The case was discussed in a multidisciplinary tumor board meeting, and the possibilities of a primary colonic malignancy with pancreatic metastasis, a primary pancreatic malignancy with colonic metastases, and a double primary malignancy of the pancreas and colon were considered. A decision was made to obtain tissue from the pancreatic mass lesion, which more likely appeared to be a primary lesion, given its characteristic appearance and elevated carbohydrate antigen 19-9 levels. Endoscopic ultrasound showed a 4.8 × 4.2 cm mass lesion of mixed echogenicity with regular margins engulfing the surrounding vessels and displaying shadowing in the body of the pancreas. Fine-needle aspiration biopsy showed neoplastic glandular structures infiltrating small segments of the nerve, and a diagnosis of adenocarcinoma with intraneural and perineural infiltration was made. Immunohistochemistry performed on both the biopsies showed diffuse positive staining of tumor cells for cytokeratin 7 (CK7) and only few cells for cytokeratin 20 (CK20), thereby confirming primary pancreatic adenocarcinoma with colonic metastases (Figures 2 and 3). Fluorodeoxyglucose and positron emission tomography CT scan showed a metabolically active infiltrative mass lesion involving the distal body and tail of the pancreas, patchy metabolic activity in the peritoneum, and surprisingly no metabolic uptake in the colon. After further discussion in the multidisciplinary tumor board meeting, the decision was made to start chemotherapy with 3 weekly cycles of injection gemcitabine, injection nab-paclitaxel (albumin-bound paclitaxel), and palliation of pain with splanchnic nerve radiofrequency ablation and celiac plexus block. He was subsequently discharged and asked to follow up after 3 cycles of chemotherapy for reassessment.

## DISCUSSION

In comparison with other similar case reports on metachronous colonic lesions, our patient had synchronous colonic lesions. Colonic metastases from other organs typically present with scirrhous morphology, and the tumor tends to infiltrate the colon wall in a manner similar to gastric “linitis plastica.”^10^ Colonoscopy findings in our case were not typical of primary colonic adenocarcinoma. The atypical appearance of these lesions suggests that the mode of spread could have been externally from peritoneum to serosa and inward or via lymphatics rather than a hematogenous mode of spread.

CK7 is expressed in the epithelial cells of pancreaticobiliary, proximal gastrointestinal tract, ovary, lungs, and breast, but not in the colon. However, CK20 is expressed in all cases of colorectal carcinomas, 62% of pancreatic carcinomas, and 50% of gastric adenocarcinomas. Hence, a combination of CK7 and CK20 is very useful to distinguish metastatic colon cancer from primary colon cancer.^11^ Immunohistochemistry is clearly important in resolving the dilemma in this case as to which tumor was primary and which was metastatic.

The fact that the fluorodeoxyglucose and positron emission tomography CT scan also failed to detect these colonic lesions suggests that colonic metastases in primary pancreatic cancer may be an underdiagnosed or an under-reported entity. If abdominal imaging had been performed before colonoscopy in our case, the focus would have completely shifted to further evaluation of the pancreatic lesion, and colonoscopy might not have been performed. This highlights the fact that primary pancreatic adenocarcinoma can have silent colonic metastases, which may not be detected unless there is a high index of suspicion.

## DISCLOSURES

Author contributions: All authors contributed equally to this manuscript. BS Ramakrishna is the article guarantor.

Financial disclosure: None to report.

Informed consent was obtained for this case report.

## References

[1] Ferlay J Colombet M Soerjomataram I Estimating the global cancer incidence and mortality in 2018: GLOBOCAN sources and methods. Int J Cancer. 2019;144(8):1941–53. 30350310 10.1002/ijc.31937

[2] Howlader N Noone AM Krapcho M SEER Cancer Statistics Review, 1975–2013. Bethesda, MD: National Cancer Institute; 2016.

[3] Hughet F Muckerjee F Javle M Locally advanced pancreatic cancer: The role of definitive chemoradiotherapy. Clin Oncol. 2014;26:560–8. 10.1016/j.clon.2014.06.002 25001636

[4] Nogueira ST Pinto BL Silva EF Pancreatic cancer presenting as colonic disease: A rare case report. Int J Surg Case Rep. 2018;44:4–7. 29454229 10.1016/j.ijscr.2018.01.019 PMC5852301

[5] Woogyeong K Yedaun L Metachronous colonic metastasis from pancreatic cancer presenting as mechanical obstruction: A case report. Clin Imaging. 2015;39:699–701. 25735449 10.1016/j.clinimag.2015.01.010

[6] Inada K Shida K Noda K Metachronous colonic metastasis from pancreatic cancer seven years post-pancreatoduodenectomy. World J Gastroenterol. 2013;19:1655–8. 10.3748/wjg.v19.i10.1665 PMC3602487 23539549

[7] Bellows C Gage T T. Stark M Metastatic pancreatic carcinoma presenting as colon carcinoma. South Med J. 2009;102:748–50. 19488001 10.1097/SMJ.0b013e3181a8fad7

[8] Ogu U Bloch R Park G A rare case of metachronous skip metastasis of pancreatic cancer to the colon. Am Surg. 2012;78:342–3. 22748524

[9] Kelley KM Myer BS Berger JJ Malignant large bowel obstruction: A rare presentation of metastatic pancreatic cancer. Am Surg. 2016;82(8):e206–8. 27657570

[10] Michalopoulos A Papadopoulos V Zatagias A Metastatic breast adenocarcinoma masquerading as colonic primary. Report of two cases. Tech Coloproctol. 2004;8(Suppl 1):s135–7. 15655598 10.1007/s10151-004-0135-8

[11] Chu P Wu E Weiss LM Cytokeratin 7 and cytokeratin 20 expression in epithelial neoplasms. Mod Pathol. 2000;13(9):962–72. 11007036 10.1038/modpathol.3880175

